# Reward Expectation Differentially Modulates Global and Local Spatial Working Memory Accuracy

**DOI:** 10.3389/fpsyg.2021.744400

**Published:** 2021-10-15

**Authors:** Qingjie Zhou, Zanzan Jiang, Jinhong Ding

**Affiliations:** Beijing Key Laboratory of Learning and Cognition, College of Psychology, Capital Normal University, Beijing, China

**Keywords:** reward expectation, spatial working memory, global-level processing, local-level processing, high cognitive load

## Abstract

Although it has been suggested that reward expectation affects the performance of spatial working memory tasks, controversial results have been found in previous experiments. Hence, it is still unclear to what extent reward expectation has an effect on working memory. To clarify this question, a memory-guided saccade task was applied, in which participants were instructed to retain and reconstruct a temporospatial sequence of four locations by moving their eyes in each trial. The global- and local-level spatial working memory accuracies were calculated to determine the reward effect on the global and local level of processing in spatial working memory tasks. Although high reward expectation enhanced the encoding of spatial information, the percentage of trials in which the cued location was correctly fixated decreased with increment of reward expectation. The reconstruction of the global temporospatial sequence was enhanced by reward expectation, whereas the local reconstruction performance was not affected by reward. Furthermore, the improvements in local representations of uncued locations and local sequences were at the cost of the representation of cued locations. The results suggest that the reward effect on spatial working memory is modulated by the level of processing, which supports the flexible resource theory during maintenance.

## Introduction

Working memory plays an essential role in human adaptive behavior and lies at the core of cognitive psychology research since its birth. The limited capacity of working memory has been an important concern since the insightful research of Miller on “the magical number seven” (Miller, 1956), and it has been demonstrated that the capacity of working memory is even less than seven (Cowan, 2001; Kawasaki and Yamaguchi, 2013; Marchette et al., 2015). However, the total capacity of working memory is not fixed; it varies across individuals (Just and Carpenter, 1992; Barrett et al., 2004) and can even be improved by motivation within the same individual (Krawczyk et al., 2007; Heitz et al., 2008; Kawasaki and Yamaguchi, 2013).

As an extrinsic motivation, monetary reward is a powerful modulator of attention. The expectation of a monetary reward narrows down the scope of attention (Filetti et al., 2019), allocates cognitive resources (Wei and Kang, 2014; Su et al., 2021), and alters cognitive executive function (Qu et al., 2013). It has been proposed that reward expectation improves working memory capacity by encouraging participants to make more efforts to fulfill the working memory tasks (Gilbert and Fiez, 2004; Hopstaken et al., 2016).

However, empirical results have revealed a complex relationship between reward and working memory. Under some circumstances, reward did improve working memory performance (Gilbert and Fiez, 2004; Taylor et al., 2004; Rowe et al., 2008; Beck et al., 2010; Savine et al., 2010; Marquand et al., 2011; Kawasaki and Yamaguchi, 2013; Sandry et al., 2014; Choi et al., 2015; Hammer et al., 2015; Fairclough and Ewing, 2017; Hefer and Dreisbach, 2017; Heritage et al., 2017; Klink et al., 2017; Allen and Ueno, 2018; Anna and Anna, 2018; Thurm et al., 2018; Gaillard et al., 2019; Magis-Weinberg et al., 2019; Manga et al., 2020; Sandry and Ricker, 2020), while other studies did not find the reward effect on working memory accuracy (Pochon et al., 2002; Krawczyk et al., 2007; Beck et al., 2010; Hager et al., 2015; Infanti et al., 2017; Smith et al., 2017; Fairclough et al., 2018; Di Rosa et al., 2019). A potential factor mediating the reward effect is working memory load. For example, the reward effect was pronounced in studies of complex tasks, such as remembering 20 locations (Cho et al., 2018) or maintaining spatial and other features simultaneously (Rowe et al., 2008; Kawasaki and Yamaguchi, 2013; Klink et al., 2017; Allen and Ueno, 2018; Anna and Anna, 2018; Gaillard et al., 2019). In contrast, when the task was relatively simple, such as the eight-arm maze task (Smith et al., 2017), the influence of reward expectation on spatial working memory disappeared. Some researchers directly manipulated working memory load and/or compared performances of participants with different working memory abilities (Taylor et al., 2004; Esteban et al., 2015; Hammer et al., 2015; Thurm et al., 2018; Gaillard et al., 2019). For example, Hammer et al. (2015) required children with attention-deficit/hyperactivity disorder (ADHD) and normally developed children to complete 2-back visual working memory tasks. The behavioral results revealed the reward expectation effect on working memory performance only in the ADHD group but not in the normally developed group. It was proposed that the disappearance of the reward effect was due to the ceiling effect (Savine et al., 2010; Esteban et al., 2015; Hammer et al., 2015).

However, the reward effect is not merely mediated by working memory load (Pochon et al., 2002; Heritage et al., 2017; Fairclough et al., 2018; Gaillard et al., 2019), and participants with better working memory ability have shown the reward effect, while others did not (Thurm et al., 2018; Manga et al., 2020). Hence, working memory load is not sufficient to explain the inconsistent results of the previous studies. There are other factors involved in the relationship between reward and working memory, such as processing level (Ahmed and Fockert, 2012) and other factors (Beck et al., 2010; Savine et al., 2010; Choi et al., 2015; Hammer et al., 2015; Heritage et al., 2017; Fairclough et al., 2018; Magis-Weinberg et al., 2019).

According to the load theory, the effectiveness of voluntary attention is impaired when working memory load is high, because of exhausting cognitive resources (Lavie et al., 2004). However, according to Ahmed and Fockert (2012), this theory is valid only when the task requires relative local-level processing, while the effect reverses when global-level processing is required. Ahmed and Fockert (2012) suggested that the ability to effectively concentrate attention to relevant local visual fields is reduced when working memory load is high. In typical spatial working memory studies, increment of spatial working memory load always couples with a more localized requirement of processing. Compared with low spatial working memory load condition, the visual field in high working memory load condition is divided into relatively small areas, and attention must be constrained to a more local visual field to take in distinct representations of multiple locations (Saarinen, 1988), while the effectiveness of voluntary attention deteriorates when the working memory load is high. Thus, the increase in working memory requirement is not only a burden to the maintenance of spatial information but also weakens the encoding of spatial locations.

The interaction between the effect of reward and the effect of working memory load may reflect the unstable reward effect on working memory. The reward effect improves working memory performance by enhancing voluntary attention (Gilbert and Fiez, 2004; Pessoa, 2009), but a high working memory load undermines the precision of spatial representations (Ahmed and Fockert, 2012). Based on these findings, we hypothesized that reward enhances spatial working memory performance at the global level, but working memory performance at the local level benefits less from reward or, even worse, the reward effect vanishes.

To verify this hypothesis, we applied a sequential memory-guided saccade task under different reward conditions. Compared with other delay-match tasks, the memory-guided saccade task is more flexible and accountable (Funahashi et al., 1993) and has been widely applied in studies of spatial working memory (Funahashi et al., 1993; Park et al., 1995; Sawaguchi and Iba, 2001; Johnston and Everling, 2008; Tsujimoto and Postle, 2012). The accuracy of saccade relies on precise spatial representation (Vergilino and Beauvillain, 2001; Theeuwes et al., 2009). The memory of sequential locations consists of location and sequence information. Sequential information is organized in either time or space, which are compatible (Fischer-Baum and Benjamin, 2014). Serial-order memory is highly connected with spatial working memory, and sequence information is represented in the form of space (van Dijck et al., 2013; Antoine et al., 2018).

In this study, spatial working memory performance was assessed both at the global and local level of similarity between stimuli sequence and scan path. In addition to the saccade landing point, fixation duration was calculated as a measurement of cognitive effort devoted to the task. Eye movements reflect the status of attention (Rayner, 1978, 2009; Theeuwes et al., 2009), and fixation duration is a valid indicator of attention (Rayner et al., 2007; Papageorgiou et al., 2014). Friedman and Liebelt (1981) found that fixation duration was prolonged when the gazed object was unusual or was required to be remembered. They suggested that the prolonged fixation duration reflects additional attention allocated to the gazed object. Considering that fixation duration covers multiple cognitive procedures, such as intake of foveal information and saccade planning (Rayner, 1998; Ludwig et al., 2014), and is influenced by factors other than cognitive effort (Rayner, 1998; Ludwig et al., 2014), comparing the fixation durations of reward cue and non-cue can provide more details about the impact of reward expectation on working memory.

## Methods

### Participants

Twenty right-handed college students (10 men and 10 women) participated in this experiment. All of them had a normal or correct-to-normal vision and color vision. They were compensated after the experiment with a basic amount of money plus a bonus depending on their performance.

### Equipment and Materials

**Figure 1:**
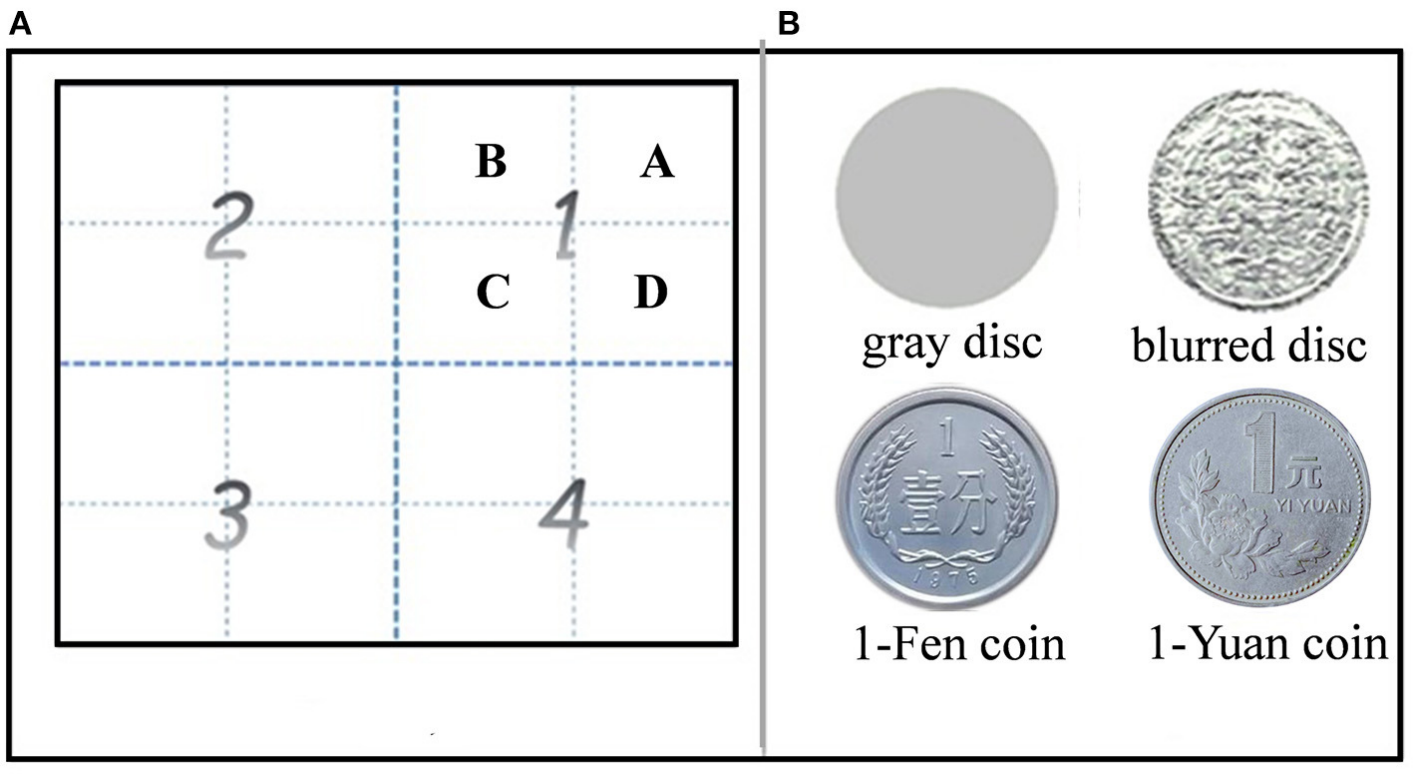
Partition of the screen for stimulus presentation **(A)** and stimuli used in the experiment **(B)**. **(A)** Four quadrants (marked by numbers) were divided into a total of 16 equal-size grids (four per quadrant, labeled by letters). **(B)** Stimuli were gray disk (top left), blurred disk (top right), 1-Fen (bottom left), and 1-Yuan (bottom right) Chinese coins.

Stimuli were presented on a 19-inch CRT monitor with a refresh rate of 120 Hz and a resolution of 1,024 × 768 pixels *via* Visual Basic. The viewing distance was 60 cm. A chin and forehead rest was used to reduce head movements. Eye positions were recorded by SMI Hi-speed eye tracker (SensoMotoric Instruments GmbH, Teltow, Germany) at a sampling rate of 350 Hz. The spatial resolution of the eye tracker was 0.1° of visual angle. A standard nine-point calibration and validation were conducted at the beginning of each block to ensure the eye data quality. Memory arrays consisted of four items, three of which were gray disks, while the fourth, a reward cue, was either a 1-Fen or 1-Yuan or blurred Chinese coin (1 Yuan equals 100 Fen in Chinese currency). The locations of reward cues in memory arrays were randomized. All stimuli were in the size of 5.59° visual angle and had the same luminance (Figure 1B). The reward expectation level was assigned to none, low, and high corresponding to reward cues of blurred, 1-Fen, and 1-Yuan Chinese coins (reward cues), respectively.

The screen was divided into 16 grids and equally distributed in four quadrants (four squares in each quadrant). Items (disks or reward cues) were displayed in squares in accordance with the experimental design.

### Procedure

A sample test paradigm was used in the experiment. During the encoding phase, a “+” appeared at the center of the screen and disappeared until the subjects fixated it steadily for at least 800 ms. Four pictures, including three gray disks and a reward cue (blurred/1-Fen/1-Yuan Chinese coins), were presented in each quadrant at one of four grids for 1,000 ms, sequentially. The stimulus positions were described by the sequential order of quadrants (denoted by bigger numbers) and the number of grids (labeled by letters; as shown in Figure 1A) in which the stimuli were displayed. The participants were asked to gaze at the pictures and to remember their locations and sequential order.

There was a delay of 800 ms before the recall phase. During the recall phase, the participants were instructed to reconstruct the spatial location sequence as precisely as possible in 5,000 ms by gazing on a blank screen. The eye scan path was described by two series of positions of different processing levels. One was the quadrant sequence, indicating the sequential order of eye positions in different quadrants, such as “1234” (denoted by letters in Figure 1A). The other one was the sequential positions at grids in each of the quadrants, denoted by letters “ABCD.” The recall performance of spatial working memory was calculated by comparing the sequences of stimulus in the encoding phase and eye scan path in the retrieval phase. Similarities of each trial were calculated according to Brandt and Stark (1997) and Eddy (2004), including global similarity (GS), which was based on the quadrant, and local similarity (LS), which was based on the grid. Both GS and LS refer to degrees of similarity between sequences of stimuli positions in the study phase and eye fixation positions in the recall phase, ranging from 0 (totally different) to 1 (the same). GS and LS indicate the recall accuracy of the global and local positions of stimuli, respectively. LS has a finer spatial scale than GS and reflects a more rigid requirement of spatial resolution.

At the end of each trial, a feedback of reward amount (money in Chinese Yuan) was shown for 1,000 ms. Coin pictures were used to indicate locations to be remembered and reward cues (Hager et al., 2015; Di Rosa et al., 2019; Manga et al., 2020). The amount of reward depended on the performance (i.e., GS and LS) and reward conditions of participants and was calculated by the formula W^*^(GS+LS)/2), where *W* = 0 for No-reward, *W* = 1 for Low-reward, and *W* = 10 for High-reward. The unit of it is the Chinese Yuan.

**Figure 2:**
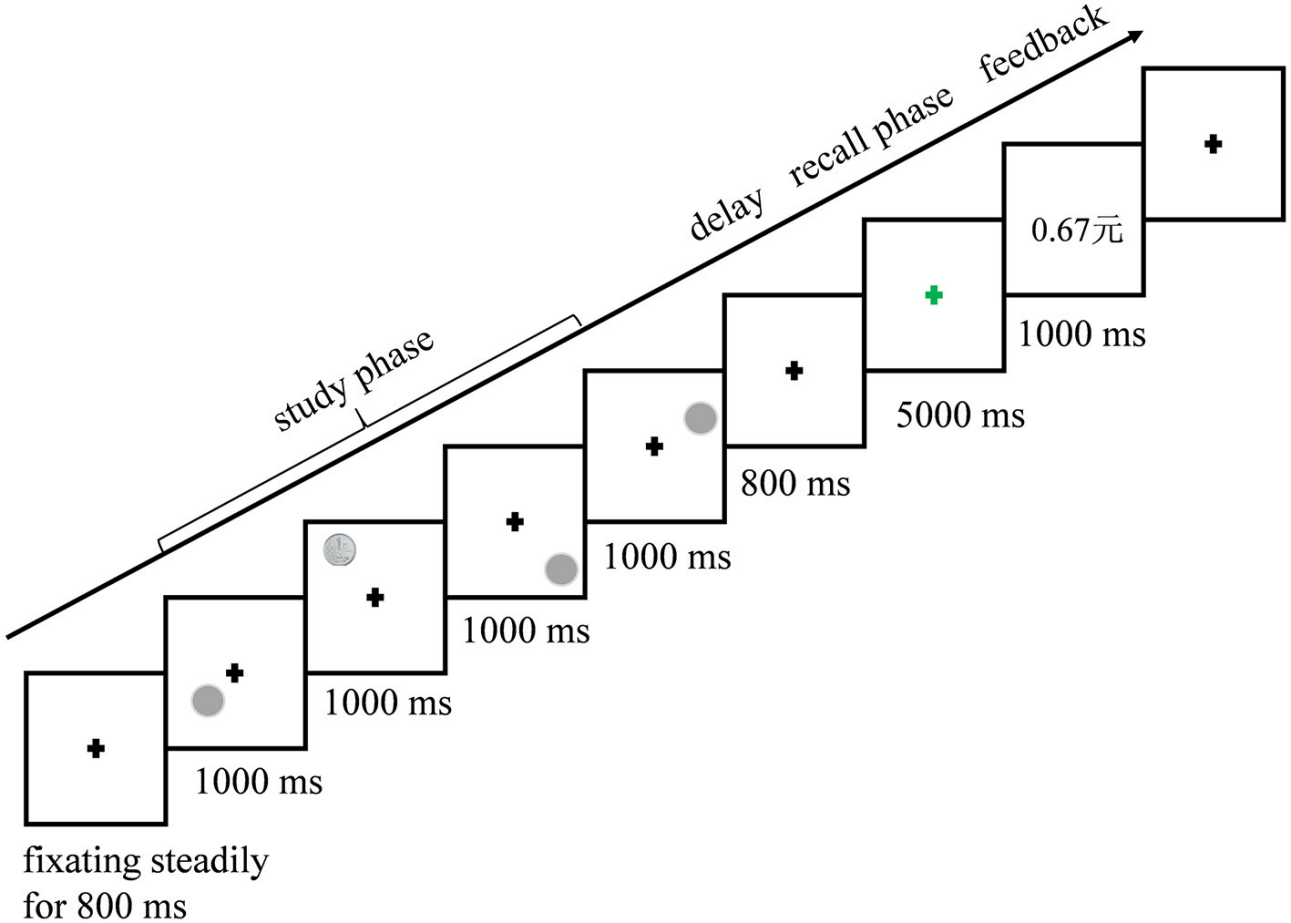
An example of the sequence of events and presentation times.

A total of 192 trials in the whole experiment were randomized and assigned into 12 blocks. There were 48 trials in each of the reward expectation conditions (No, Low, and High). The procedure of one trial is shown in Figure 2.

### Data Preprocessing

Dependent variables in the present research were derived from the fixation data. Fixations were defined using a temporal threshold of 100 ms and a spatial threshold of 2° visual angle, which were calculated offline. Then, the mean fixation number and the mean fixation duration were generated and grouped by the reward expectation conditions and location of fixation. The percentage of trials in which the cued grid was correctly re-fixed was used as the index of reward-cue memory performance. The spatial working memory task performance was evaluated by the sequence similarity proposed by Eddy (2004). Specifically, locations with the same temporal order of stimuli in the study phase and of eye fixation in the recall phase were compared, and each pair of overlapped locations scored 1 point. Sequence similarity was calculated by dividing the total score of four pairs of locations by 4, with a range from 0 (totally wrong) to 1 (totally correct).

## Results

### Eye Fixations During the Study Phase

**Figure 3:**
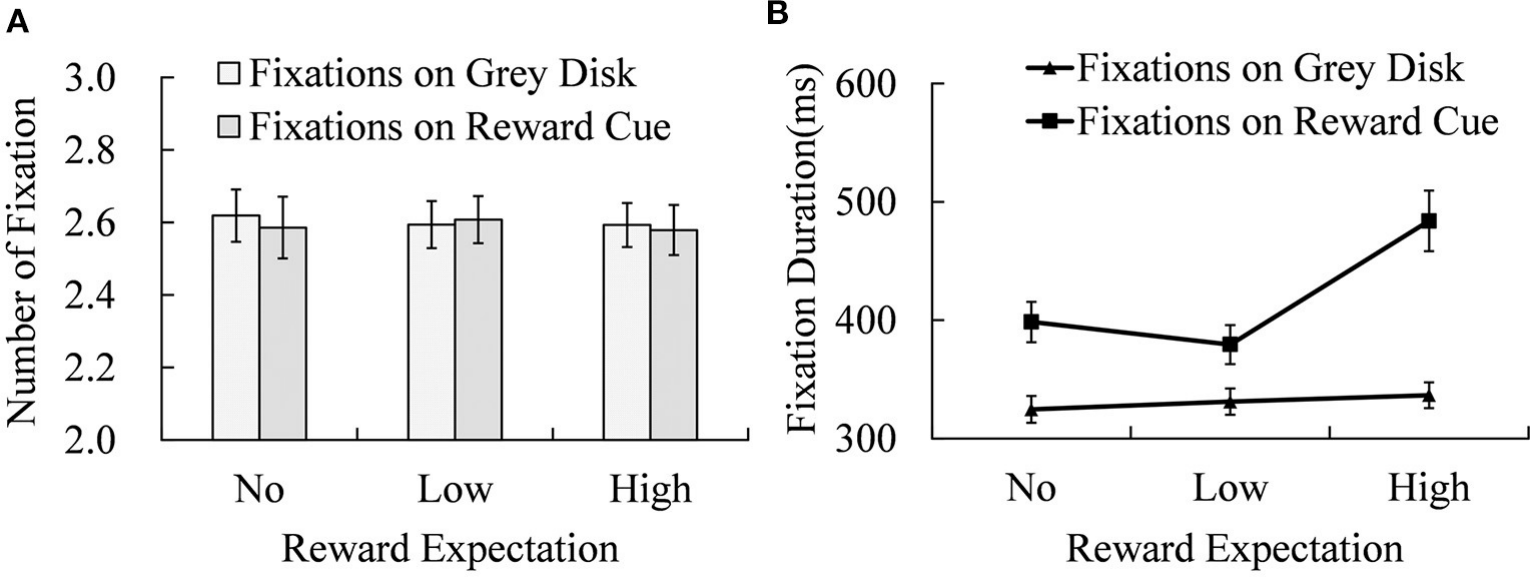
The numbers of fixation **(A)** and mean fixation duration **(B)** on cue and non-cue under different reward expectation conditions. Error bars indicate the standard error of mean (SEM).

During the study phase, three gray disks and a reward cue were presented sequentially one by one. The subjects were instructed to remember their spatial and temporal locations. We segregated the numbers of eye fixations and their durations by whether they were on the cue or non-cue (gray disk; as shown in Figure 3).

There was no significant effect of reward expectation or fixation position (on non-cue or cue) on the number of fixations. However, the fixation duration was significantly affected by reward expectation [*M*_No−reward_ = (362 ± 14) ms; *M*_Low−reward_ = (355 ± 13) ms; *M*_High−reward_ = (410 ± 17) ms; *F*_(2, 38)_ = 25.736, *p* < 0.001, $\eta_{\text{p}}^{2}$ = 0.575], fixation position [*M*_cue_ = (421 ± 17) ms; *M*_disk_ = (331 ± 11) ms; *F*_(1, 19)_ = 91.351, *p* < 0.001, $\eta_{\text{p}}^{2}$ = 0.828], and their interaction [*F*_(2, 38)_ = 15.087, *p* < 0.001, $\eta_{\text{p}}^{2}$ = 0.443]. Simple effect analysis showed significant differences in fixation duration between the fixations on locations of reward cue and those on locations of non-cue disk under No-reward [*t*_(19)_ = 8.714, *p* < 0.001, Cohen's *d* = 1.949], Low-reward [*t*_(19)_ = 4.810, *p* < 0.001, Cohen's *d* = 1.075], and High-reward expectation conditions [*t*_(19)_ = 7.005, *p* < 0.001, Cohen's *d* = 1.566]. Reward expectation effects were significant for fixation durations of both reward cue [*F*_(2, 38)_ = 6.020, *p* < 0.001, $\eta_{\text{p}}^{2}$ = 0.241] and non-cue disks [*F*_(2, 38)_ = 23.120, *p* < 0.001, $\eta_{\text{p}}^{2}$ = 0.549]. For the non-cue disks, fixation durations of High-reward cue were significantly longer than those of Low- [*t*_(19)_ = 2.174, *p* = 0.043, Cohen's *d* = 0.486] and No-reward cues [*t*_(19)_ = 4.077, *p* < 0.001, Cohen's *d* = 0.912]. The difference in fixation durations between No-reward and Low-reward cues was marginally significant [*t*_(19)_ = 2.057, *p* = 0.054, Cohen's *d* = 0.460]. When reward cues were fixated, the same pattern appeared. Fixation durations of High-reward cues were significantly greater than those of No-reward [*t*_(19)_ = 4.266, *p* < 0.001, Cohen's *d* = 0.954] and Low-reward cues [*t*_(19)_ = 5.596, *p* < 0.001, Cohen's *d* = 1.251], and there was no significant difference between No- and Low-reward cues.

### Eye Fixations on the Location of Reward Cue During the Retrieval Phase

**Figure 4:**
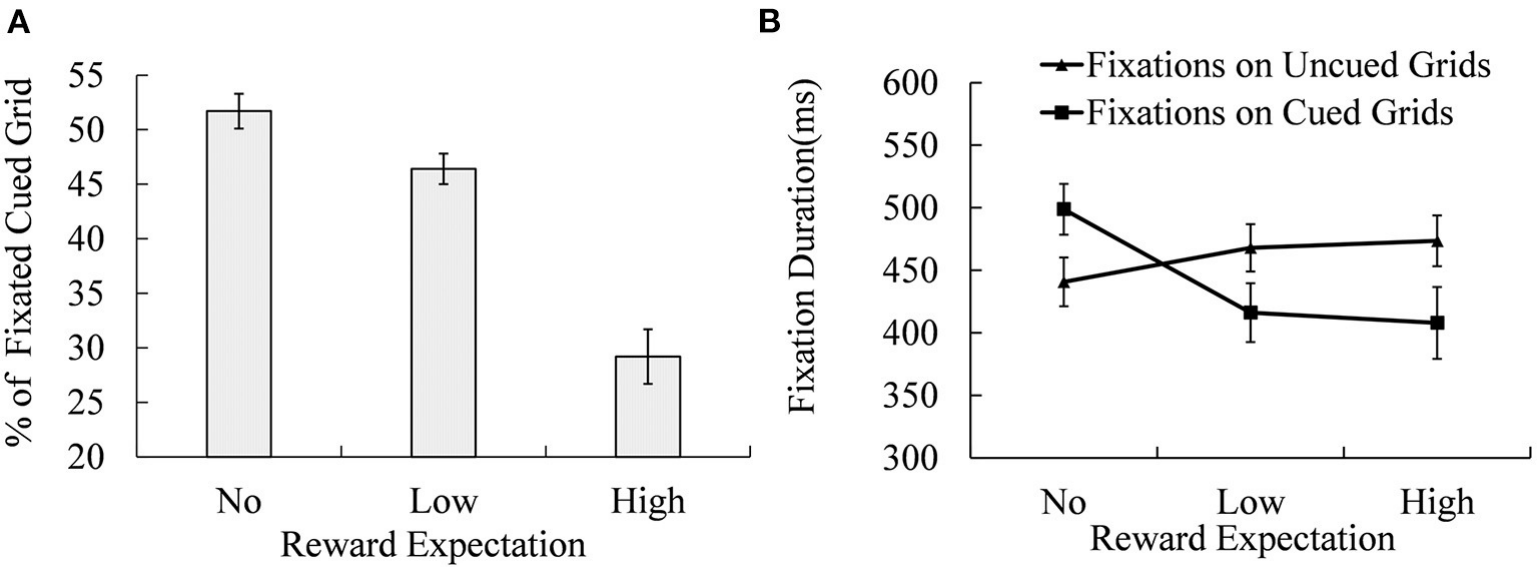
Percentage of the fixated cued locations **(A)** and the fixation durations of both the cued grids were fixated and unfixated **(B)** under different reward expectation conditions. Error bars indicate the standard error of mean (SEM).

During the retrieval phase, the percentage of cued grids that were correctly fixated (as shown in Figure 4A) and the fixation durations of fixated or unfixated cued grids under different reward expectation conditions were calculated (as shown in Figure 4B).

Analysis of variance (ANOVA) of the percentage of trials in which cued grids were fixated (as shown in Figure 4A) revealed a significant effect of reward expectation [*M*_No−reward_ = 51.70% ± 1.60%; *M*_Low−reward_ = 46.40% ± 1.40%; *M*_High−reward_ = 29.20% ± 2.50%; *F*_(2, 38)_ = 44.800, *p* < 0.001, $\eta_{\text{p}}^{2}$ = 0.702]. Multiple comparisons showed that the No-reward cued grid was fixated more frequently than the Low-reward [*t*_(19)_ = 2.731, *p* < 0.05, Cohen's *d* = 0.886] and High-reward cued grids [*t*_(19)_ = 9.461, *p* < 0.001, Cohen's *d* = 3.070], while the Low-reward cued grid received more fixations than the High-reward cued grid [*t*_(19)_ = 5.702, *p* < 0.001, Cohen's *d* = 1.850].

A 2 (fixation on cued grid and non-cued grid) × 3 (No-, Low-, and High-reward cue) repeated-measures ANOVA of fixation duration revealed a significant effect of fixation location [*M*_cued_ = (441 ± 22) ms; *M*_uncued_ = (461 ± 19) ms; *F*_(1, 19)_ = 6.798, *p* = 0.017, $\eta_{\text{p}}^{2}$ = 0.263], reward expectation [*M*_No−reward_ = (470 ± 19) ms; *M*_Low−reward_ = (442 ± 21) ms; *M*_High−reward_ = (441 ± 23) ms; *F*_(2, 38)_ = 5.808, *p* = 0.019, $\eta_{\text{p}}^{2}$ = 0.234], and their interaction [*F*_(2, 38)_ = 22.793, *p* < 0.001, $\eta_{\text{p}}^{2}$ = 0.545]. Simple effect analysis showed significant differences in fixation durations between cued and non-cued grids under No- [*F*_(1, 19)_ = 33.280, *p* < 0.001, $\eta_{\text{p}}^{2}$ = 0.637], Low- [*F*_(1, 19)_ = 23.640, *p* < 0.001, $\eta_{\text{p}}^{2}$ = 0.554], and High-reward expectation conditions [*F*_(1, 19)_ = 11.910, *p* < 0.001, $\eta_{\text{p}}^{2}$ = 0.385]. Reward expectation effects were significant for mean fixation durations on both cued grid [*F*_(2, 38)_ = 11.710, *p* < 0.010, $\eta_{\text{p}}^{2}$ = 0.381] and non-cued grid [*F*_(2, 38)_ = 15.11, *p* < 0.001, $\eta_{\text{p}}^{2}$ = 0.443]. Duration of fixation on non-cued grid of No-reward expectation was significantly shorter than that of Low-reward [*t*_(19)_ = −5.051, *p* < 0.001, Cohen's *d* = 1.639] or High-reward expectation conditions [*t*_(19)_ = −3.717, *p* = 0.001, Cohen's *d* = 1.206]. There was no significant difference in fixation duration between High- and Low-reward expectations. For the cued grid, the fixation duration of No-reward expectation was significantly longer than that of High- [*t*_(19)_ = 6.715, *p* < 0.001, Cohen's *d* = 2.179] and Low-reward expectation conditions [*t*_(19)_ = 3.825, *p* = 0.001, Cohen's *d* = 1.241], and there was no significant difference between High- and Low- reward expectation conditions.

### The Retrieval Performance of Spatial Working Memory Task

**Figure 5:**
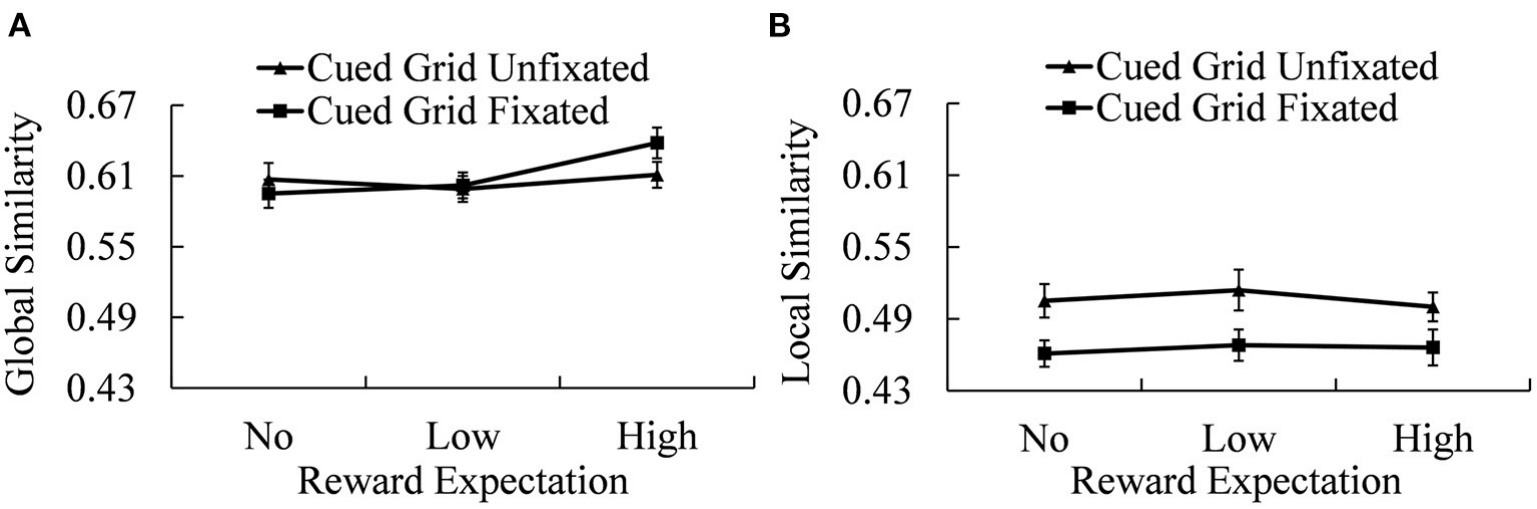
Recall scores of GS **(A)** and LS **(B)** under different reward expectation conditions. Error bars indicate the standard error of mean (SEM).

Spatial working memory performances (both GS and LS between the sequence of stimuli positions and eye fixation positions in the recall phase) under different conditions are shown in Figure 5.

For the GS, a 2 (cued grid fixated and unfixated) × 3 (No-, Low-, and High-reward cue) repeated-measures ANOVA revealed a significant effect of reward expectation [*M*_No−reward_ = 0.601 ± 0.012; *M*_Low−reward_ = 0.601 ± 0.010; *M*_High−reward_ = 0.625 ± 0.011; *F*_(2, 38)_ = 7.078, *p* = 0.002, $\eta_{\text{p}}^{2}$ = 0.271]. The interaction between the cued grid and reward expectation is also significant [*F*_(2, 38)_ = 4.356, *p* = 0.020, $\eta_{\text{p}}^{2}$ = 0.187]. Simple effect analysis revealed a significant reward expectation effect when the locations of reward were successfully re-fixated [*F*_(2, 38)_ = 12.095, *p* < 0.001, $\eta_{\text{p}}^{2}$ = 0.389] but not when the locations of reward cue were not re-fixated. Further analysis showed that, when the locations of reward cue were successfully re-fixated, there were significant differences in GS between the Low-reward condition and the High-reward condition [*t*_(19)_ = 3.788, *p* = 0.002, Cohen's *d* = 0.847] and between the No-reward condition and the High-reward condition [*t*_(19)_ = 4.611, *p* < 0.001, Cohen's *d* = 1.031]. However, there was no significant difference between the No-reward condition and the Low-reward condition. The difference of GS between conditions when the cued grid was fixated and when the cued grid was unfixated was significant in High-reward expectation condition [*t*_(19)_ = 2.813, *p* = 0.011, Cohen's *d* = 0.629], but not in the other two reward expectation conditions (*p* > 0.100).

Local similarity showed a different statistical pattern, that is, 2 (cued grid fixated and unfixated) × 3 (No-, Low-, and High-reward cue) repeated-measures ANOVA revealed a significant main effect of re-fixation [*M*_unfixated_ = 0.521 ± 0.012; *M*_fixated_ = 0.465 ± 0.010; *F*_(2, 38)_ = 26.413, *p* < 0.001, $\eta_{\text{p}}^{2}$ = 0.582], showing that when the cued grids failed to be re-fixated, scores of local similarity went up. Other effects on LS were not significant.

## Discussion

The results of the current research showed that reward expectation affected the encoding and maintenance of temporally organized spatial representations. Four-location temporal sequence was encoded into the working memory, and more attention was paid to locations with high rewards in the study phase. These spatial location representations were maintained in the delay phase and retrieved successively in the reconstruction phase.

### Reward Expectation Enhances Voluntary Attention in the Encoding Phase

The results of the study phase showed that stimuli, reward cues or not, in the High-reward expectation condition were fixated longer than those in No- and Low-reward expectation conditions (as shown in Figure 5). Considering the effect of reward expectation on recall performance in this study and the indecisive relationship between pure fixation duration and working memory performance in previous studies (Saint-Aubin et al., 2007; Oi et al., 2015), the changed fixation duration in the study phase of this study likely reflect differences in attention allocation under different reward expectation conditions. Specifically, the participants paid more attention to the task when reward expectation was high, compared with the No-reward and Low-reward conditions.

The result of fixation duration in the study phase is in line with previous studies that reported that reward expectation prompted encoding of working memory (Wallis et al., 2015; for a review, see Botvinick and Braver, 2015; Klink et al., 2017; Roberts et al., 2017). In their first experiment, Wallis et al. (2015) found that reward improved encoding of reward-associated items, and the reward effect was generalized to other items in the memory list. In this research, fixation durations on gray disks were prolonged in High-reward conditions as well. It seems that the mechanism underlying the reward effect in the encoding phase is more general than the specified encoding of the reward cue. A plausible explanation is that reward expectation enhances arousal, which in turn provides more cognitive resources for working memory encoding (Murray, 2007; Murayama and Kitagami, 2014; Unsworth and Robison, 2015), and participants are willing to make more efforts to obtain a higher reward. The null result of fixation number and the longer fixation duration of reward cue in the study phase are predictable. The design of the task in this research restricted the saccadic patterns of participants in the study phase and led to equivalent fixations under all reward expectation levels. These reward cues contained additional reward information compared with gray disks, requiring additional processes.

### Reward Effects on Different Processing Levels

The main aim of this research was to explore the reward effect on spatial working memory at different processing levels. As predicted, we found a significant reward effect on GS, the indicator of global processing, but no reward effect on local similarity, the indicator of local processing. Furthermore, the percentage of trials in which the cued grid was correctly re-fixated during the retrieval phase, a relatively local indicator, decreased with the increment of reward. High reward improved spatial working memory performance at the global level but undermined the precision of spatial representations at the local level.

Mean fixation duration in the recall phase is in line with this conclusion. The mean fixation duration in memory-guided saccade tasks is affected by the strength of the memory trace, and it requires more time and effort to recall a weak memory trace (Burke et al., 2012; Haj et al., 2017; Dang et al., 2021). Therefore, when the memory of the next saccade target is weak, prolonged fixation duration is required to generate the following saccade (Meghanathan et al., 2020). Eye-tracking data of this research showed that fixation on uncued locations in the recall phase, sometimes followed by a saccade to the cued location, prolonged as the reward expectation was higher. A potential explanation is that, in this research, the memory for the cued location was degraded when the reward expectation was high, and more time was required before the saccade to the cued location could be generated.

The results of similarities at different processing levels and the negative reward effect on the representation of cued location are consistent with the hypothesis made by Ahmed and Fockert (2012), that is, the authors suggested that working memory load modulates selective attention to different levels of the same stimulus. When working memory load is high, information at a more global level is easily selected, while local-level information is ignored. It is difficult to constrain attention to the local level with a high working memory load. As a result, reward expectation prompted global similarity but did not affect local similarity in this study.

### Modulation of Sustained Control on Spatial Working Memory Representation

An interesting finding of this research is the trade-off between different indicators. As mentioned above, the mean fixation duration of the recall phase reflects degraded representations of cued locations and promoted representations of uncued locations with the increment of reward. Local similarities of trials in which the cued location was falsely re-fixated were higher than local similarities of trials in which the cued location was successfully re-fixated. It seems that the improvement of representations of uncued locations and local similarities comes at the cost of representation of cued locations.

These results of cued-location memory are consistent with the proposal of flexible attention theory (Sandry et al., 2014; Sandry and Ricker, 2020), which suggests that the cognitive system assigns attention resources flexibly among items in working memory, and the elevation of working memory performance of a certain item is at the cost of performance of other items (Sandry et al., 2014; Allen and Ueno, 2018; Sandry and Ricker, 2020). In this research, to obtain a higher reward, goal-directed cognitive control may have inhibited the maintenance of reward-cue location and allocated saved resources to the maintenance of other locations in the delay phase. Moreover, higher local similarities were observed in the trials in which the cued location was falsely re-fixated.

### Limitations of This Study

During the study phase of this study, all three conditions (No-, Low-, and High-) of the reward cue were randomly presented at different temporal positions. It may lead to a confounding effect. The different intervals of processing the reward cue mean that the levels of processing or the motivational states may vary accordingly. Specifically, motivational states might be identical in the three reward conditions until the presentation of the reward cue. Moreover, according to Sandry and Ricker (2020), the temporal position does affect performance in working memory tasks. Hence, the temporal position of reward cues should be considered in future studies.

## Conclusion

By applying sequential memory-guided eye movement tasks, we reached the following conclusions regarding the reward effect on spatial working memory: (a) reward expectation enhances the encoding of spatial locations by improving voluntary attention, and (b) reward affects reconstruction only at the global level but not at the local level, in which the cognitive resource is reallocated among reward- and non-reward-associated items in working memory to maximize the reward effect.

## Data Availability Statement

The raw data supporting the conclusions of this article will be made available by the authors, without undue reservation.

## Ethics Statement

The studies involving human participants were reviewed and approved by Psychological Ethics Committee of Capital Normal University. The patients/participants provided their written informed consent to participate in this study.

## Author Contributions

JD and QZ discussed and developed the idea and wrote the introduction, methods, results, and discussion. ZJ designed the study with input from JD and QZ and ran the experiment on all participants. JD and ZJ were highly involved in the analysis of the results. All authors contributed to the article and approved the submitted version.

## Conflict of Interest

The authors declare that the research was conducted in the absence of any commercial or financial relationships that could be construed as a potential conflict of interest.

## Publisher's Note

All claims expressed in this article are solely those of the authors and do not necessarily represent those of their affiliated organizations, or those of the publisher, the editors and the reviewers. Any product that may be evaluated in this article, or claim that may be made by its manufacturer, is not guaranteed or endorsed by the publisher.
